# Guanidinium 3-nitro­benzoate

**DOI:** 10.1107/S160053681002581X

**Published:** 2010-07-07

**Authors:** Graham Smith, Urs D. Wermuth

**Affiliations:** a Faculty of Science and Technology, Queensland University of Technology, GPO Box 2434, Brisbane, Queensland 4001, Australia; b School of Biomolecular and Physical Sciences, Griffith University, Nathan, Queensland 4111, Australia

## Abstract

The title compound, CH_6_N_3_^+^·C_7_H_4_NO_4_^−^, an anhydrous guanidinium salt, shows a N—H⋯O hydrogen-bond network in which the guanidinium cation is involved in three cyclic *R*_2_^1^(6) hydrogen-bonding associations with separate carboxyl­ate O-atom acceptors. Further peripheral associations include a cyclic *R*_1_^2^(4) cation–anion inter­action, forming inter­linked undulating sheets in the three-dimensional structure.

## Related literature

For the structures of other guanidinium benzoate salts, see: Kleb *et al.* (1998 ▶); Pereira Silva *et al.* (2007 ▶, 2010 ▶). For graph-set analysis, see: Etter *et al.* (1990 ▶).
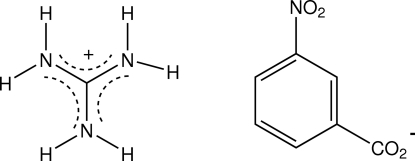


## Experimental

### 

#### Crystal data

CH_6_N_3_^+^·C_7_H_4_NO_4_^−^
*M* *_r_* = 226.20
Orthorhombic, 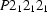

*a* = 7.3978 (12) Å
*b* = 10.1302 (12) Å
*c* = 13.7118 (17) Å
*V* = 1027.6 (2) Å^3^
*Z* = 4
Mo *K*α radiation
μ = 0.12 mm^−1^
*T* = 297 K
0.30 × 0.30 × 0.20 mm

#### Data collection

Oxford Diffraction Gemini-S CCD-detector diffractometer
Absorption correction: multi-scan (*CrysAlis PRO*; Oxford Diffraction, 2009 ▶) *T*_min_ = 0.94, *T*_max_ = 0.98
7455 measured reflections
1252 independent reflections
1092 reflections with *I* > 2σ(*I*)
*R*_int_ = 0.030

#### Refinement

*R*[*F* ^2^ > 2σ(*F* ^2^)] = 0.035
*wR*(*F* ^2^) = 0.096
*S* = 1.03
1252 reflections
169 parameters
H atoms treated by a mixture of independent and constrained refinement
Δρ_max_ = 0.15 e Å^−3^
Δρ_min_ = −0.16 e Å^−3^

### 

Data collection: *CrysAlis PRO* (Oxford Diffraction, 2009 ▶); cell refinement: *CrysAlis PRO*; data reduction: *CrysAlis PRO*; program(s) used to solve structure: *SIR92* (Altomare *et al.*, 1994 ▶); program(s) used to refine structure: *SHELXL97* (Sheldrick, 2008 ▶) within *WinGX* (Farrugia, 1999 ▶); molecular graphics: *PLATON* (Spek, 2009 ▶); software used to prepare material for publication: *PLATON*.

## Supplementary Material

Crystal structure: contains datablocks global, I. DOI: 10.1107/S160053681002581X/bv2146sup1.cif

Structure factors: contains datablocks I. DOI: 10.1107/S160053681002581X/bv2146Isup2.hkl

Additional supplementary materials:  crystallographic information; 3D view; checkCIF report

## Figures and Tables

**Table 1:** Hydrogen-bond geometry (Å, °)

*D*—H⋯*A*	*D*—H	H⋯*A*	*D*⋯*A*	*D*—H⋯*A*
N1*G*—H11*G*⋯O12	0.82 (3)	2.48 (3)	3.161 (3)	142 (3)
N1*G*—H12*G*⋯O12^i^	0.83 (3)	2.09 (3)	2.887 (3)	162 (3)
N2*G*—H21*G*⋯O11^i^	0.91 (3)	2.42 (3)	3.292 (3)	160 (2)
N2*G*—H21*G*⋯O12^i^	0.91 (3)	2.43 (3)	3.159 (3)	137 (2)
N2*G*—H22*G*⋯O11^ii^	0.86 (3)	2.29 (3)	3.020 (3)	143 (3)
N3*G*—H31*G*⋯O11^ii^	0.86 (3)	1.97 (3)	2.783 (3)	157 (3)
N3*G*—H32*G*⋯O12	0.91 (3)	1.90 (3)	2.794 (3)	166 (3)

Symmetry codes: (i) 
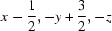
; (ii) 
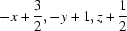
.

**Fig. 1.**
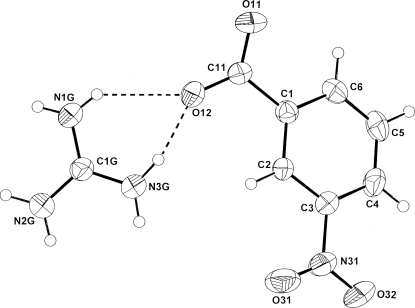
The molecular configuration and atom-numbering scheme for the cation and anion species in (I). Non-H atoms are shown as 40% probability ellipsoids. Inter-ion hydrogen bonds are shown as dashed lines.

**Fig. 2.**
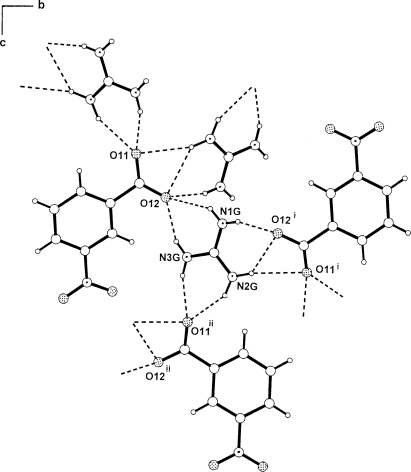
Peripheral hydrogen-bonding extension of the R21(6)- associated guanidinium-tris(3-nitrobenzoate) structures of (I), viewed down the a cell direction. For symmetry codes, see Table 1. Hydrogen bonds are shown as dashed lines.

**Fig. 3.**
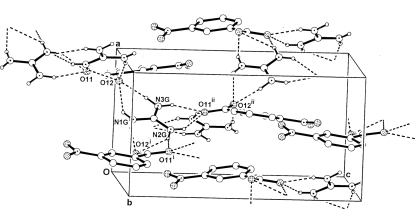
The three-dimensional structure of (I) viewed down the approximate b cell direction. Non-associative H atoms are omitted.

## supplementary crystallographic information

### Comment

The structures of the guanidinium salts of the simple benzoic acids are not numerous in the crystallographic literature, being limited to the benzoate (Pereira Silva *et al.*, 2007), 4-aminobenzoate (Pereira Silva *et al.*, 2010) and 4-nitrobenzoate (Kleb *et al.*, 1998). In these anhydrous structures and those of the anhydrous guanidinium salts of aromatic carboxylates generally, the cations give variously cyclic hydrogen-bonding interactions which may be classified by the graph sets *R*^2^_2_(8), *R*_1_^2^(4) or *R*_2_^1^(6) (Etter *et al.*, 1990). Our 1:1 stoichiometric reaction of 3-nitrobenzoic acid with guanidinium carbonate in methanol gave large relatively hard, chemically stable crystals of guanidinium 3-nitrobenzoate, CH_6_N_3_^+^ C_7_H_4_NO_4_^-^ (I), and the structure is reported here.

In the structure of (I) each guanidinium cation is involved in three cyclic *R*_2_^1^(6) hydrogen-bonding associations (Table 1) with separate carboxylate O-acceptors (Figs. 1, 2). Further peripheral associations include a cyclic *R*_1_^2^(4) cation–anion interaction, form inter-linked undulating sheets which give a three-dimensional framework structure (Fig. 3).

The carboxylate group of the anion is rotated slightly out of the plane of the benzene ring [torsion angle C2–C1–C11–O11, 160.0 (2)°]. However, the unassociated nitro group is essentially coplanar with the ring [torsion angle C2–C3–N31–O32, 174.4 (2)°].

### Experimental

The title compound was synthesized by heating together under reflux for 10 minutes 1 mmol of 3-nitrobenzoic acid and 0.5 mmol of guanidine carbonate in 50 ml of methanol. After concentration to *ca* 30 ml, partial room temperature evaporation of the hot-filtered solution gave large colourless plates (m.p. 514 K) from which a suitable analytical specimen was cleaved.

### Refinement

Guanidinium hydrogen atoms were located by difference methods and their positional and isotropic displacement parameters were refined. The H atoms of the aromatic ring of the anion were included in the refinement in calculated positions (C–H = 0.93 Å) and allowed to ride, with *U*_iso_(H) = 1.2*U*_eq_(C). Friedel pairs were merged in the data set used for final structure refinement.

### Crystal data

**Table:**

CH_6_N_3_^+^·C_7_H_4_NO_4_^−^	*D*_x_ = 1.462 Mg m^−^^3^
*M**_r_* = 226.20	Melting point: 514 K
Orthorhombic, *P*2_1_2_1_2_1_	Mo *K*α radiation, λ = 0.71073 Å
Hall symbol: P 2ac 2ab	Cell parameters from 2964 reflections
*a* = 7.3978 (12) Å	θ = 3.0–28.9°
*b* = 10.1302 (12) Å	µ = 0.12 mm^−^^1^
*c* = 13.7118 (17) Å	*T* = 297 K
*V* = 1027.6 (2) Å^3^	Block, colourless
*Z* = 4	0.30 × 0.30 × 0.20 mm
*F*(000) = 472	

### Data collection

**Table:**

Oxford Diffraction Gemini-S CCD-detector diffractometer	1252 independent reflections
Radiation source: Enhance (Mo) X-ray source	1092 reflections with *I* > 2σ(*I*)
graphite	*R*_int_ = 0.030
ω scans	θ_max_ = 26.5°, θ_min_ = 3.0°
Absorption correction: multi-scan (*CrysAlis PRO*; Oxford Diffraction, 2009)	*h* = −9→9
*T*_min_ = 0.94, *T*_max_ = 0.98	*k* = −12→11
7455 measured reflections	*l* = −17→17

### Refinement

**Table:**

Refinement on *F*^2^	Primary atom site location: structure-invariant direct methods
Least-squares matrix: full	Secondary atom site location: difference Fourier map
*R*[*F*^2^ > 2σ(*F*^2^)] = 0.035	Hydrogen site location: inferred from neighbouring sites
*wR*(*F*^2^) = 0.096	H atoms treated by a mixture of independent and constrained refinement
*S* = 1.03	*w* = 1/[σ^2^(*F*_o_^2^) + (0.0716*P*)^2^] where *P* = (*F*_o_^2^ + 2*F*_c_^2^)/3
1252 reflections	(Δ/σ)_max_ < 0.001
169 parameters	Δρ_max_ = 0.15 e Å^−^^3^
0 restraints	Δρ_min_ = −0.15 e Å^−^^3^

### Special details

**Table:**

Geometry. Bond distances, angles *etc*. have been calculated using the rounded fractional coordinates. All su's are estimated from the variances of the (full) variance-covariance matrix. The cell e.s.d.'s are taken into account in the estimation of distances, angles and torsion angles
Refinement. Refinement of *F*^2^ against ALL reflections. The weighted *R*-factor *wR* and goodness of fit *S* are based on *F*^2^, conventional *R*-factors *R* are based on *F*, with *F* set to zero for negative *F*^2^. The threshold expression of *F*^2^ > σ(*F*^2^) is used only for calculating *R*-factors(gt) *etc*. and is not relevant to the choice of reflections for refinement. *R*-factors based on *F*^2^ are statistically about twice as large as those based on *F*, and *R*- factors based on ALL data will be even larger.

### Fractional atomic coordinates and isotropic or equivalent isotropic displacement parameters (Å^2^)

**Table:**

	*x*	*y*	*z*	*U*_iso_*/*U*_eq_	
O11	0.8853 (3)	0.39642 (19)	−0.15616 (11)	0.0763 (7)	
O12	0.8391 (3)	0.51830 (14)	−0.02410 (12)	0.0587 (5)	
O31	0.9478 (4)	0.2929 (2)	0.29189 (13)	0.0891 (8)	
O32	0.8919 (4)	0.0871 (2)	0.30563 (13)	0.0879 (8)	
N31	0.9085 (3)	0.1871 (2)	0.25680 (14)	0.0582 (6)	
C1	0.8552 (3)	0.28643 (18)	−0.00456 (13)	0.0395 (5)	
C2	0.8834 (3)	0.29418 (19)	0.09621 (14)	0.0395 (5)	
C3	0.8802 (3)	0.1785 (2)	0.15053 (15)	0.0438 (6)	
C4	0.8496 (3)	0.0567 (2)	0.10846 (18)	0.0546 (7)	
C5	0.8224 (3)	0.0507 (2)	0.00897 (18)	0.0579 (8)	
C6	0.8262 (3)	0.1633 (2)	−0.04688 (16)	0.0487 (6)	
C11	0.8592 (3)	0.4098 (2)	−0.06632 (14)	0.0472 (6)	
N1G	0.5698 (4)	0.7518 (2)	0.02372 (14)	0.0596 (7)	
N2G	0.5023 (3)	0.7924 (2)	0.18458 (16)	0.0624 (7)	
N3G	0.6559 (3)	0.6050 (2)	0.14213 (17)	0.0599 (7)	
C1G	0.5763 (3)	0.7166 (2)	0.11723 (14)	0.0468 (6)	
H2	0.90390	0.37520	0.12620	0.0470*	
H4	0.84730	−0.01950	0.14630	0.0660*	
H5	0.80130	−0.03050	−0.02060	0.0690*	
H6	0.80920	0.15720	−0.11390	0.0580*	
H11G	0.617 (4)	0.701 (3)	−0.015 (2)	0.070 (9)*	
H12G	0.514 (4)	0.821 (3)	0.012 (2)	0.072 (8)*	
H21G	0.444 (4)	0.869 (3)	0.170 (2)	0.077 (9)*	
H22G	0.506 (4)	0.767 (3)	0.244 (2)	0.065 (8)*	
H31G	0.666 (4)	0.588 (3)	0.203 (2)	0.065 (8)*	
H32G	0.715 (4)	0.563 (3)	0.093 (2)	0.073 (9)*	

### Atomic displacement parameters (Å^2^)

**Table:**

	*U*^11^	*U*^22^	*U*^33^	*U*^12^	*U*^13^	*U*^23^
O11	0.1174 (16)	0.0759 (12)	0.0357 (8)	0.0071 (13)	0.0050 (10)	0.0078 (8)
O12	0.0866 (12)	0.0375 (7)	0.0520 (8)	−0.0007 (8)	0.0034 (9)	0.0074 (6)
O31	0.148 (2)	0.0728 (12)	0.0464 (9)	−0.0228 (14)	−0.0197 (12)	0.0015 (9)
O32	0.1360 (19)	0.0687 (11)	0.0591 (11)	0.0044 (13)	0.0017 (12)	0.0305 (9)
N31	0.0728 (11)	0.0565 (11)	0.0452 (10)	0.0004 (11)	−0.0036 (9)	0.0113 (8)
C1	0.0411 (9)	0.0384 (10)	0.0390 (9)	−0.0016 (9)	0.0000 (9)	−0.0014 (8)
C2	0.0476 (10)	0.0313 (8)	0.0395 (9)	−0.0005 (9)	−0.0007 (8)	0.0007 (7)
C3	0.0486 (10)	0.0413 (10)	0.0415 (10)	0.0011 (9)	−0.0006 (9)	0.0044 (8)
C4	0.0643 (13)	0.0340 (10)	0.0656 (13)	−0.0007 (10)	0.0029 (12)	0.0083 (10)
C5	0.0679 (15)	0.0348 (10)	0.0710 (15)	−0.0073 (10)	0.0012 (13)	−0.0151 (10)
C6	0.0527 (11)	0.0490 (11)	0.0445 (10)	−0.0037 (10)	0.0000 (9)	−0.0100 (9)
C11	0.0597 (12)	0.0444 (10)	0.0375 (10)	0.0004 (10)	0.0016 (10)	0.0038 (9)
N1G	0.0873 (16)	0.0448 (10)	0.0467 (12)	0.0052 (11)	0.0022 (11)	0.0093 (9)
N2G	0.0885 (15)	0.0519 (11)	0.0469 (11)	0.0123 (12)	0.0033 (11)	−0.0015 (9)
N3G	0.0854 (14)	0.0511 (11)	0.0433 (10)	0.0130 (12)	0.0016 (11)	0.0069 (9)
C1G	0.0588 (12)	0.0398 (10)	0.0418 (11)	−0.0032 (10)	−0.0027 (9)	0.0025 (9)

### Geometric parameters (Å, °)

**Table:**

O11—C11	1.254 (2)	N3G—H32G	0.91 (3)
O12—C11	1.251 (3)	C1—C6	1.392 (3)
O31—N31	1.210 (3)	C1—C11	1.510 (3)
O32—N31	1.221 (3)	C1—C2	1.400 (3)
N31—C3	1.475 (3)	C2—C3	1.389 (3)
N1G—C1G	1.332 (3)	C3—C4	1.381 (3)
N2G—C1G	1.320 (3)	C4—C5	1.380 (3)
N3G—C1G	1.320 (3)	C5—C6	1.374 (3)
N1G—H12G	0.83 (3)	C2—H2	0.9300
N1G—H11G	0.82 (3)	C4—H4	0.9300
N2G—H22G	0.86 (3)	C5—H5	0.9300
N2G—H21G	0.91 (3)	C6—H6	0.9300
N3G—H31G	0.86 (3)		
			
O31—N31—O32	122.8 (2)	C2—C3—C4	122.2 (2)
O31—N31—C3	118.64 (19)	C3—C4—C5	118.4 (2)
O32—N31—C3	118.60 (19)	C4—C5—C6	120.8 (2)
C1G—N1G—H12G	115.5 (19)	C1—C6—C5	121.0 (2)
H11G—N1G—H12G	128 (3)	O11—C11—C1	117.67 (18)
C1G—N1G—H11G	116 (2)	O12—C11—C1	117.73 (17)
H21G—N2G—H22G	119 (3)	O11—C11—O12	124.6 (2)
C1G—N2G—H21G	122.6 (18)	C3—C2—H2	121.00
C1G—N2G—H22G	119 (2)	C1—C2—H2	121.00
H31G—N3G—H32G	126 (3)	C3—C4—H4	121.00
C1G—N3G—H32G	115.1 (19)	C5—C4—H4	121.00
C1G—N3G—H31G	118 (2)	C4—C5—H5	120.00
C6—C1—C11	120.72 (17)	C6—C5—H5	120.00
C2—C1—C11	120.29 (17)	C5—C6—H6	120.00
C2—C1—C6	118.99 (17)	C1—C6—H6	119.00
C1—C2—C3	118.66 (18)	N2G—C1G—N3G	120.2 (2)
N31—C3—C4	119.27 (19)	N1G—C1G—N2G	120.2 (2)
N31—C3—C2	118.53 (18)	N1G—C1G—N3G	119.6 (2)
			
O31—N31—C3—C2	−5.8 (3)	C2—C1—C11—O12	−18.9 (3)
O31—N31—C3—C4	174.9 (2)	C6—C1—C11—O11	−19.1 (3)
O32—N31—C3—C2	174.4 (2)	C6—C1—C11—O12	162.0 (2)
O32—N31—C3—C4	−4.9 (3)	C1—C2—C3—N31	−179.5 (2)
C6—C1—C2—C3	−0.4 (3)	C1—C2—C3—C4	−0.2 (3)
C11—C1—C2—C3	−179.5 (2)	N31—C3—C4—C5	179.6 (2)
C2—C1—C6—C5	1.0 (3)	C2—C3—C4—C5	0.4 (3)
C11—C1—C6—C5	−180.0 (2)	C3—C4—C5—C6	0.2 (3)
C2—C1—C11—O11	160.0 (2)	C4—C5—C6—C1	−0.8 (3)

### Hydrogen-bond geometry (Å, °)

**Table:**

*D*—H···*A*	*D*—H	H···*A*	*D*···*A*	*D*—H···*A*
N1G—H11G···O12	0.82 (3)	2.48 (3)	3.161 (3)	142 (3)
N1G—H12G···O12^i^	0.83 (3)	2.09 (3)	2.887 (3)	162 (3)
N2G—H21G···O11^i^	0.91 (3)	2.42 (3)	3.292 (3)	160 (2)
N2G—H21G···O12^i^	0.91 (3)	2.43 (3)	3.159 (3)	137 (2)
N2G—H22G···O11^ii^	0.86 (3)	2.29 (3)	3.020 (3)	143 (3)
N3G—H31G···O11^ii^	0.86 (3)	1.97 (3)	2.783 (3)	157 (3)
N3G—H32G···O12	0.91 (3)	1.90 (3)	2.794 (3)	166 (3)
C4—H4···O31^iii^	0.93	2.57	3.355 (3)	142

Symmetry codes: (i) *x*−1/2, −*y*+3/2, −*z*; (ii) −*x*+3/2, −*y*+1, *z*+1/2; (iii) −*x*+2, *y*−1/2, −*z*+1/2.
